# Persistence of Pristine Deep-Sea Coral Gardens in the Mediterranean Sea (SW Sardinia)

**DOI:** 10.1371/journal.pone.0119393

**Published:** 2015-03-19

**Authors:** Marzia Bo, Giorgio Bavestrello, Michela Angiolillo, Lucio Calcagnile, Simonepietro Canese, Rita Cannas, Alessandro Cau, Marisa D’Elia, Filippo D’Oriano, Maria Cristina Follesa, Gianluca Quarta, Angelo Cau

**Affiliations:** 1 Università degli Studi di Genova, Dipartimento di Scienze della Terra, dell'Ambiente e della Vita, Genova, Italy; 2 Istituto Superiore per la Protezione e la Ricerca Ambientale, Roma, Italy; 3 Università del Salento, Centro di Datazione e Diagnostica, Dipartimento di Ingegneria dell’Innovazione, Lecce, Italy; 4 Università di Cagliari, Dipartimento di Scienze della Vita e dell'Ambiente, Cagliari, Italy; 5 Geophi S.r.l. c/o Research Area Consiglio Nazionale delle Ricerche, Bologna, Italy; Dauphin Island Sea Lab, UNITED STATES

## Abstract

*Leiopathes glaberrima* is a tall arborescent black coral species structuring important facies of the deep-sea rocky bottoms of the Mediterranean Sea that are severely stifled by fishing activities. At present, however, no morphological *in vivo* description, ecological characterization, age dating and evaluation of the possible conservation actions have ever been made for any population of this species in the basin. A dense coral population was reported during two Remotely Operated Vehicle (ROV) surveys conducted on a rocky bank off the SW coasts of Sardinia (Western Mediterranean Sea). *L*. *glaberrima* forms up to 2 m-tall colonies with a maximal observed basal diameter of nearly 7 cm. The radiocarbon dating carried out on a colony from this site with a 4 cm basal diameter revealed an approximately age of 2000 years. Considering the size-frequency distribution of the colonies in the area it is possible to hypothesize the existence of other millennial specimens occupying a supposedly very stable ecosystem. The persistence of this ecosystem is likely guaranteed by the heterogeneous rocky substrate hosting the black coral population that represents a physical barrier against the mechanical impacts acted on the surrounding muddy areas, heavily exploited as trawling fishing grounds. This favorable condition, together with the existence of a nursery area for catsharks within the coral ramifications and the occurrence of a meadow of the now rare soft bottom alcyonacean *Isidella elongata* in small surviving muddy enclaves, indicates that this ecosystem have to be considered a pristine Mediterranean deep-sea coral sanctuary that would deserve special protection.

## Introduction

In the terrestrial environment, trees form complex ecosystems, the forests, which represent important three-dimensional habitats supporting high levels of biodiversity both within their area and in their neighborhoods [1]. Forests supply food, protection, and support to a great variety of organisms, which may establish occasional or highly specialized relationships with both the dead and living portions of the trees [1].

Similarly, oceans host extraordinary examples of forest-like ecosystems, internationally identified as gardens, comparable in complexity, biodiversity, and structuring role to the terrestrial ones, but, especially in the deep-sea, entirely structured by colonial animals [2, 3, 4]. In the Mediterranean Sea, the habitat-forming species of these ecosystems are various colonial anthozoans, such as some alcyonaceans and black corals, which typically show an arborescent vertical development and may inhabit vast areas of rocky bottoms [5, 2, 4, 6, 7, 8]. The flexible organic skeletons of some alcyonaceans and black corals offer a weaker resistance to the current, allowing them to live in areas with a higher hydrodynamism [9, 10, 11, 12], and, at the same time, may potentially create local turbulence conditions among the ramifications thus favoring the persistence of food in suspension [13, 14, 3].

The existence of Mediterranean deep-sea black coral gardens is well known due to the fact that these species are common in the fishing bycatch [15, 16] and some of them have been sporadically subjected in the past to collection for the jewellery trade [17, 18]. However, it does not yet exist a comprehensive mapping of the populations of these species, which have always been reported, due to their deep bathymetric distribution, as rare [19]. Recent ROV surveys have documented numerous Mediterranean black coral gardens occupying rocky cliffs or shoals where they may form very abundant monospecific populations, such as those of *Antipathella subpinnata* (Ellis & Solander, 1786) and *Parantipathes larix* (Esper, 1790) [2, 12]. Mixed populations of black corals and other habitat-forming anthozoans have been observed both along the continental platform [20, 8] and among the bathyal white corals communities up to 500 m depth [21, 22], while only sparse colonies have been recently reported in bathyal habitat along French canyons [23].

Another aspect that these marine ecosystems share with the terrestrial ones is the longevity of the structuring species [24]. Similarly to the oldest terrestrial trees, also some coral species may live for thousands of years [25, 26]. Among the most longevous marine species known so far there are two hexacorallians genera (the zoanthid *Savalia* and the antipatharian *Leiopathes*) characterized by a proteinaceous and chitinous skeleton and reaching millennial ages (up to 2742 years and 4265 years, respectively, for two deep-sea Pacific specimens) [25, 27] (see Table 1 for a summary on *Leiopathes* sp.).

**Table 1:** Summary of the radiocarbon dating literature data for *Leiopathes* specimens.

Sample	Age (years)	Growth rate (μm y^-1^)	Diameter (mm)	Site	Depth (m)	Reference
(BC)#5	2320*	5–13	23.2	Hawaii	450	Roark et al., 2006
Leio 1	4265*	2–4	11.8	Hawaii	400–500	Roark et al., 2009
Leio 2	295*	2	1.4	Hawaii	400–500	Roark et al., 2009
A9902	198°	14.5	5.8	Georgia	679	Williams et al., 2006
A8601	483^†^	nd	14	Florida	593	Williams et al., 2006
A8401	290^†^	nd	8.3	Florida	561	Williams et al., 2006
AG002	386^†^	nd	11.2	Gulf Mexico	307	Williams et al., 2006
GOM-TOW-BC1	530*	22	15.2	Gulf Mexico	304	Prouty et al., 2011
GOM-TOW-BC2	1680*	8	22.8	Gulf Mexico	304	Prouty et al., 2011
GOM-JSL05–4876-BC1	670*	17	16.2	Gulf Mexico	312	Prouty et al., 2011
GOM-JSL09–3728-BC1	890*	14	28.6	Gulf Mexico	317	Prouty et al., 2011
GOM-JSL04–4734-BC1	2100*	8	36.1	Gulf Mexico	310	Prouty et al., 2011
DOP-1985	2320*	6.6–7.2	33.1	Azores	366	Carreiro-Silva et al., 2013
DOP-1356	395*	18–25	17.8	Azores	307	Carreiro-Silva et al., 2013
DOP-4588	550*	22–25	26	Azores	366	Carreiro-Silva et al., 2013
DOP-799	275*	18–33	14.2	Azores	293	Carreiro-Silva et al., 2013
DOP-4587	335*	3.9–7.0	5.2	Azores	366	Carreiro-Silva et al., 2013
LTL12302	1973*	nd	40	Mediterranean Sea	200	Present study

* Values obtained through ^14^C dating

° Values obtained through ^210^Pb dating

^†^ Age values extrapolated based on growth rings.

With the exception of the specimens from the Gulf of Mexico, showing a linear growth trend [26], most of the analyzed *Leiopathes* specimens showed a differential radial growth in time. Some studies found fastest initial rates (13 μm per year), which tend to progressively slow down (5 μm per year) [25, 27], while others found slow initial and final growth rates (~4 to 5 μm per year) separated by a period of more rapid growth (20 μm yr^−1^) [28]. Growth rates seem not related to the colony height or basal diameter, while may vary among specimens and in accordance to numerous other parameters, such as locality and depth [27, 28]. Extreme longevity, in any case, remains the common character found in all the populations studied so far and this discovery has triggered the use of *Leiopathes* species as biological archives of paleoclimatic information and environmental indicators [29, 30, 31, 32].

The aim of this paper was to characterize the deep-sea coral community of the Carloforte Shoal (Southwestern Sardinia) studying, for the first time, the population structure of the dominant species, the black coral *Leiopathes glaberrima* (Esper, 1788). This large, arborescent species, despite it was described over two centuries ago in the Mediterranean Sea and may form vast populations starting from 200 m depth [11, 15], is one of the least known. *L*. *glaberrima* represents the type species of the Family Leiopathidae [33]. The genus *Leiopathes*, the only one of the family, comprehends 8 species [33, 34] of which four have been reported from the Indian Ocean (*Leiopathes secunda* Opresko, 1998, *Leiopathes bullosa* Opresko, 1998 and *Leiopathes acanthophora* Opresko, 1998 *Leiopathes valdiviae* (Pax, 1915)), three are known exclusively from the North Atlantic Ocean (*Leiopathes montana* Molodstova, 2011, *Leiopathes expansa* Johnson, 1899, *Leiopathes grimaldii* Roule, 1902), while *L*. *glaberrima* is the only one reported, so far, both in the North Atlantic Ocean (Macaronesian Archipelago and Bay of Biscay) and in the Mediterranean basin. Firstly described from the Gulf of Naples [35], this species has been recently reported in numerous localities of both the western and eastern basins from 100 to 720 m depth, from the Alboran Sea to Crete [36, 37, 38, 17, 39, 40, 41, 21, 23, 22, 15, 42].

In this paper we elucidated the *in vivo* morphological characteristics of *L*. *glaberrima*, its habitat preferences and its associated fauna in order to define the ecological role of this species. Data have been interpreted also in a conservation key, by quantifying the fishing impact on both the rocky and surrounding soft bottom communities and through the age analysis of a black coral specimen.

## Materials and Methods

### Ethics statement

The ROV Campaign was authorized by Italian Coast Guard (Compamare Cagliari). The radiocarbon dating was conducted on a colony of *L*. *glaberrima* collected as bycatch of fishing in the area of the Carloforte Shoal and preserved in the collection of the DISVA (University of Cagliari).

### Study area

**Fig 1:**
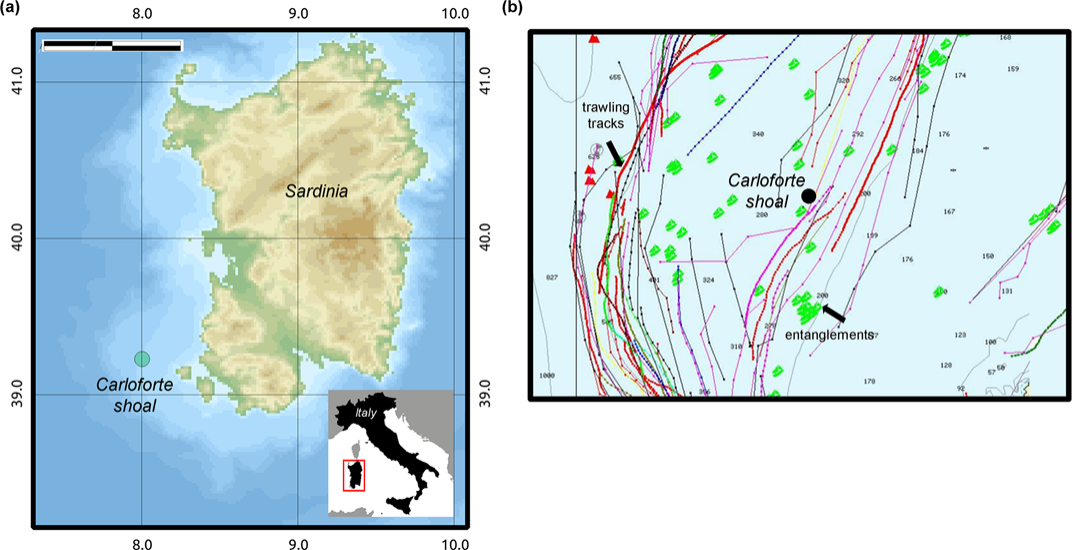
The Carloforte Shoal (NW Sardinia, Western Mediterranean Sea). **(a)** Location of the study area. **(b)** VMS tracks of trawlers in the study area and location of the investigated shoal (black dot). The other symbols on the map represent areas of entanglements for fishermen’s gears (possibly rocky elevations). The shoal is within a free-trawling zone.

The study area, denominated the Carloforte Shoal is located 11 nautical miles off the Southwestern coasts of S. Pietro, a volcanic island comprehended in the Sulcis Archipelago (western Mediterranean Sea) (Fig. 1a). The Carloforte Shoal is situated within a complex topographic region in which numerous rocky elevations emerge from a flat muddy bottoms at about 210 m depth. This region is surrounded by an area subjected to intense trawling and long-lines activities as evidenced by the VMS (Vessel Monitoring System) data sheet (Fig. 1b), such that the rocky bottoms and their immediate nearby soft bottoms are avoided from being repeatedly worked.

**Fig 2:**
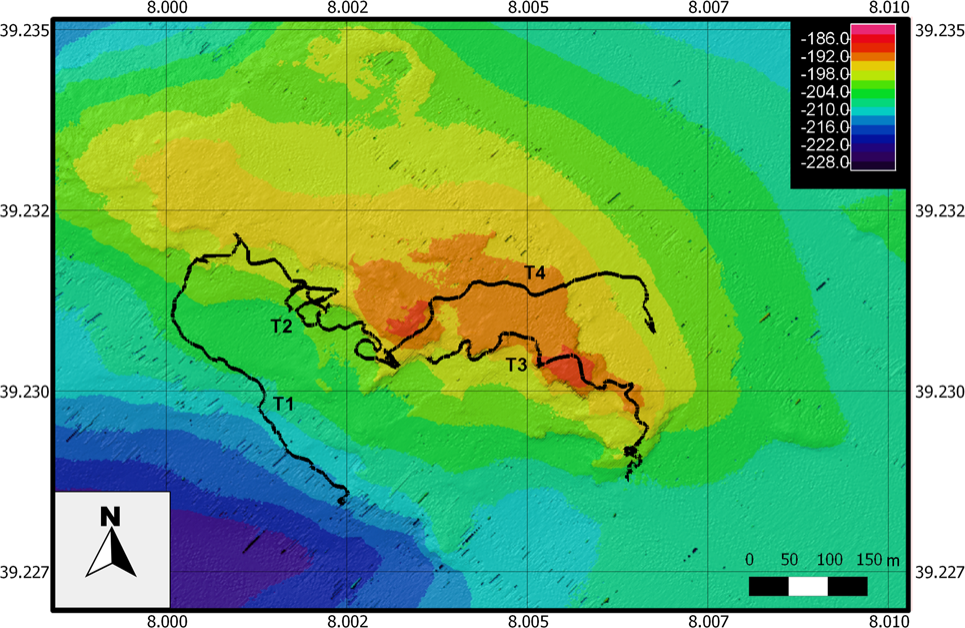
High-resolution bottom topography. Multibeam maps of the studied shoal. The four ROV transects are represented in the map (T1, 650 m long, muddy area; T2, 380 m long and T3, 520 m long, bench terraces area, T4, 460 m long, plateau area).

The investigated shoal is an oblong rocky elevation, about 0.5 km^2^ in surface, with a minimum depth of 186 m, encountered along the Southwestern side of the structure (Fig. 2). This area of the shoal is characterized by a series of bench terraces only slightly covered by sediments, while, towards the eastward side, the shoal continues with a rocky plateau with only few rocky boulders emerging from patches of detritic sand. At about 200–210 m depth, a belt of muddy bottom is observed surrounding the shoal (Fig. 2).

In terms of hydrodynamism, the Southwestern part of Sardinia, where the Carloforte Shoal is located, is found in an area characterized by strong mesoscale cyclonic eddies and a recent three-dimensional numerical model, studying the surface mean circulation of this area, put in evidence a costal upwelling of deep waters along these coasts, as supported by SST (Sea Surface Temperature) satellite imagery [43].

### Data acquisition and statistical analyses

The high resolution morpho-bathymetric map (Fig. 2) was obtained by using a hull mounted MultiBeam echosounder, the EM 2040 Kongsberg operating with 300 KHz of frequency. Data were acquired with 40% lateral overlap and processed to remove spikes due to navigation system problems and/or to the acquisition system. Acquisition and processing of data were performed using the CARIS packet (CARIS HIPS and SIPS 8.1.2, Canada). The final data were organized in a grid with cell size of 1 m using the geographic system WGS84 UTM32 N.

Video and photo footage was gathered by a ROV “Pollux” during two surveys conducted in October 2011 and September 2013 on board of the Research Vessel *Astrea* exploring the Carloforte Shoal in a depth range comprehended between 186 and 210 m depth. ROV was equipped with a digital camera (Nikon D80, 10 megapixel), a strobe (Nikon SB 400), a high definition video camera (Sony HDR-HC7), a navigation camera (1/3-inch SONY CCD, focal length 4–9 mm), and 3 jaw grabbers. The ROV hosted also a depth sensor, a compass, and three laser beams providing a 10-cm scale for the measurement of the frames area and size of organisms. The ROV was equipped with an underwater acoustic tracking position system (Tracklink 1500 MA, LinkQuest Inc.) providing detailed records of the tracks along the seabed.

Four video transects were carried out on the Carloforte Shoal in three distinct habitats: 1) mud (transect T1, 650 m long), 2) bench terraces (transects T2 and T3, 380 and 520 m long, respectively), and 3) plateau (transect T4, 460 m long) (Fig. 2). Seven hundred and twenty-seven frames (166, 451, and 110 frames for the three habitats, respectively) were randomly extrapolated from the video footage with the software DVDVideoSoft for a total of about 2110 m^2^ of explored area (frames on average 3 m^2^). Each frame was then elaborated with the software ImageJ (Rasband W., Research Services Branch, Maryland, USA) in order to calculate the density of each target species (N° colonies m^-2^ ± Standard Error). Target species were considered as all the arborescent, habitat-forming anthozoans including scleractinians, alcyonaceans, antipatharians and zoanthids.

ROV-Imaging technique was employed also to define the morphometric characteristics of the black coral colonies (basal diameter, height and width) in order study the population structure of *L*. *glaberrima*. Measures of height and width were gathered also for *Isidella elongata*. Other information, such as associated fauna and the *in vivo* aspect of the black coral colonies, has been registered. Finally, the fishing impact, based on the number of frames showing lost gears (N° impacted frames) and their direct damages on the colonies (N° of entangled colonies) [11], has been quantitatively estimated.

Portions of five colonies of *L*. *glaberrima* were collected in the study area and preserved in 4% formaldehyde and ethanol 95° for morphological analyses together with samples collected in two other localities (Table 2). The cnidome of the species was examined by squeezing polyps or their portions (mouth, tentacles and interpolypar coenenchyme) on a slide and measuring, by means of an ocular reticle, the cnidocysts (length and width in μm, mean, standard deviation, and coefficient of variation, n = 50) under optical microscopy (Dialuz 20EB, Leitz, 100x oil immersion, total magnification 1000x). For the SEM analysis, fragments of the ramifications were coated with gold-palladium in a Balzer Union evaporator and examined with a Philips EM 515 SEM.

**Table 2:** Summary table of the specimens used for the morphological and histological analyses.

Sample code	Date	Depth	Locality	Habitat
LEIO	25.5.2010	209 m	Palinuro Seamount, S Tyrrhenian Sea	Rocky shelf along Seamount flanks
SARD1	23.10.2011	200 m	Carloforte Shoal, Sardinia	Rocky bench terraces
SARD2	23.10.2011	200 m	Carloforte Shoal, Sardinia	Rocky bench terraces
LIGU7	6.6.2012	208 m	Vedove Shoal, Liguria	Rocky shoal
SARD19a	4.9.2013	190 m	Carloforte Shoal, Sardinia	Rocky bench terraces
SARD19b	4.9.2013	190 m	Carloforte Shoal, Sardinia	Rocky bench terraces
SARD19c	4.9.2013	190 m	Carloforte Shoal, Sardinia	Rocky bench terraces

A one-way ANOSIM was carried out to test for differences in species relative abundance between the assemblages of the three investigated areas of the Carloforte Shoal (BT, bench terraces; M, mud; P, plateau). The entire data set was considered: data, sqr(x)transformed, tested with a Bray-Curtis similarity measure, were distributed homogenously with n = 451, 166, and 110 frames, respectively, for BT, M, and P. Moreover, to find out whether there was a significant difference in relative abundance between the three areas of the bank for the dominant target species and for the total coral abundance, a Kruskal-Wallis test was performed (with p = probability, H = Kruskal-Wallis statistic). These analyses were performed using PAST for Windows version 1.91 [44].

Data of height, width and basal diameter of *L*. *glaberrima* (n = 155), normally distributed (checked with the Shapiro-Wilk test, p<0.001), were used to apply, with Excel, two linear regression analyses and were tested with a two-tailed Pearson’s correlation. Data of height and width of *I*. *elongata* (n = 109), not normally distributed, were tested with a Spearman’s correlation.

Size-frequency distribution curves were also obtained to express the size structure of the populations of *L*. *glaberrima* and *I*. *elongata* (in terms of height classes of the colonies).

### Radiocarbon dating

One colony of *L*. *glaberrima* with a 4 cm basal diameter and a 20 cm long stem (ahead of the rupture) recently collected as fishery bycatch in the area of the Carloforte Shoal and preserved in the collection of DISVA, University of Cagliari, was used for radiocarbon dating. Analyses were carried out by AMS (Accelerator Mass Spectrometry) at CEDAD (Centre for Dating and Diagnostics), University of Salento, Italy [45].

**Fig 3:**
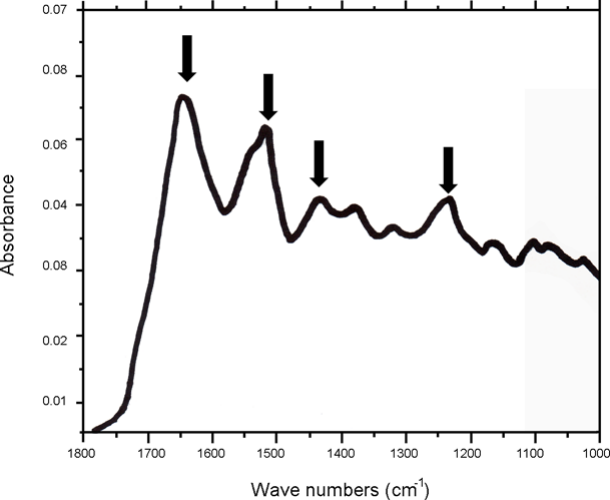
ATR-FTIR spectrum (FTIR spectrometer in attenuated total reflection (ATR) mode) obtained from the organic fraction extracted from one of the samples. The position of the characteristics IR absorption bands of collagen are indicated by arrows as reference.

Two sub-samples were selected for the analyses from the core of the base and the apex of the specimen (Table 3). Selected samples were submitted to standard processing procedures [46] at the CEDAD chemical laboratories aimed at the extraction of the organic fraction suitable for dating, at the removal of any exogenous contamination and the conversion of the samples into a form suitable to be measured with the AMS spectrometer. The selected samples were crushed to powder in a mortar, demineralized by 1% HCl at room temperature and gelatinized in acidified water (HCl, pH = 3) at 85°C. Gelatine was then filtered on 0.45 μm pore silver filters [46]. In order to chemically characterize the extracted fraction a sub-sample was obtained from each sample and submitted to FTIR (Fourier Transform Infrared) analyses that were carried out at the Chemistry-Physics laboratory of the University of Salento by using a Perkin Elmer Spotlight FTIR spectrometer in attenuated total reflection (ATR) mode. Fig. 3 shows the ATR-FTIR spectrum obtained for one of the samples (similar ones were obtained for all the analyzed samples). In the spectrum sharp and intense IR absorption peaks corresponding to collagen are evidenced (arrows in Fig. 3) allowing assessing that the extracted fraction is mainly composed by collagen [47]. The obtained collagen was then combusted to carbon dioxide in sealed quartz tubes together with CuO and silver wool and finally reduced to graphite at 600°C by using hydrogen as reducing agent and iron powder as catalyst [48]. The extracted graphite (1 mg ca) was then pressed in the target holders of the accelerator mass spectrometer for the measurement of ^14^C/^12^C and ^13^C/^12^C isotopic ratios. From the measured isotopic ratios conventional radiocarbon ages were then calculated according to [49].

**Table 3:** List of the samples submitted to AMS radiocarbon dating.

Sampling position	Laboratory Code	Radiocarbon age	Calibrated age (68.2%) probability
Apex-Inner part	LTL12302A	2144 ± 40 BP	290 ± 60 AD
Base-Inner Part	LTL12302C	2356 ± 40 BP	40 ± 55 AD

Conventional radiocarbon ages were then calibrated to calendar years by using the MARINE09 calibration curve valid for marine data [50] and the OxCal Vers. 4.0 Software [51]. A local reservoir correction ΔR = 58±15 years was used as average value for the Mediterranean Sea [52].

## Results

### The community of the Carloforte Shoal

**Fig 4:**
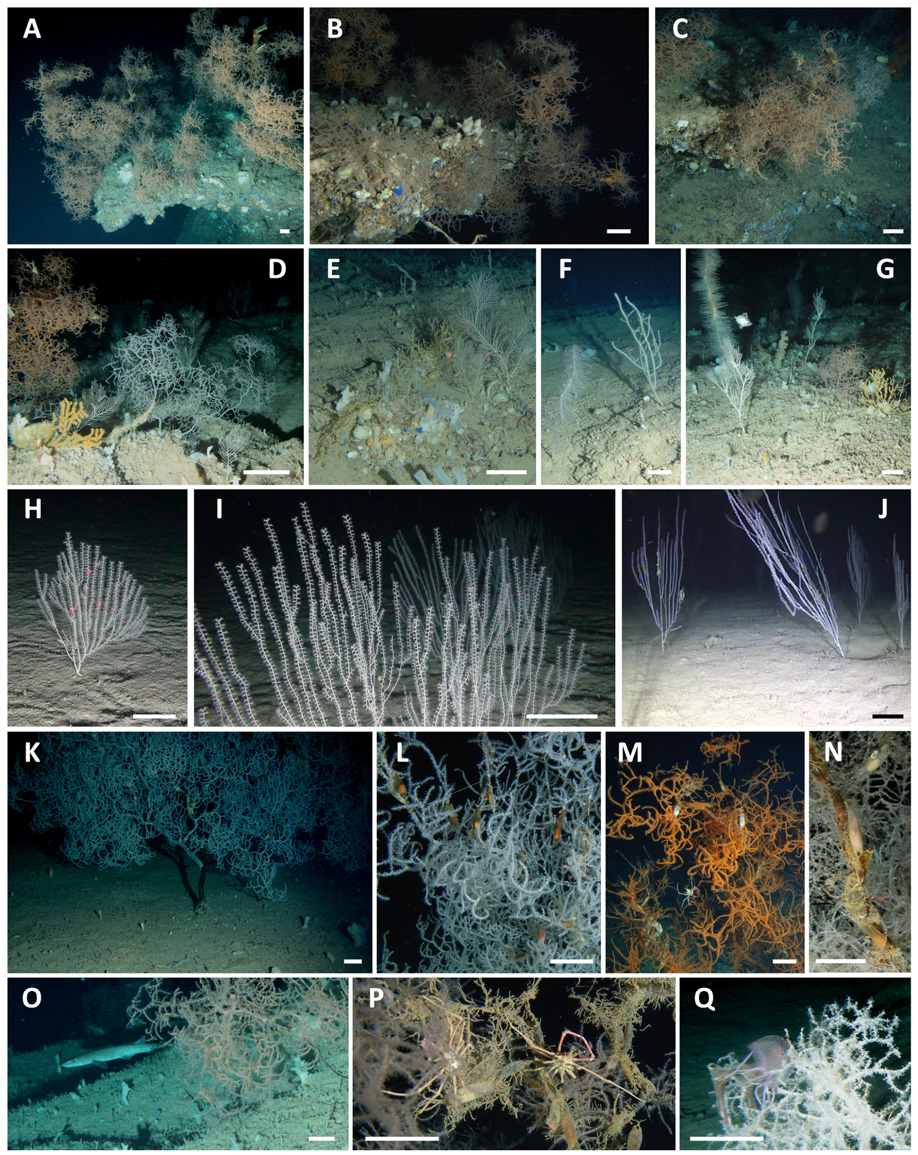
ROV images of the coral community of the Carloforte Shoal. **A-C.** Arborescent colonies of *Leiopathes glaberrima* densely arranged on the rocky bench terraces. **D.** Coral assemblage of a rocky emergence of the plateau. **E.** Colonies of *Callogorgia verticillata* surrounded by *Bebryce mollis* on the plateau. **F.** The black corals *Parantipathes larix* and *Antipathes dichotoma* on the silted bottom of the plateau. **G.** Mixed assemblage of black corals and alcyonaceans on the plateau. **H-J.** Colonies of *Isidella elongata* on the soft bottom around the rocky areas. **K.** Giant specimen of *L*. *glaberrima* anchored on a flat, silted rocky bottom. **L-N.** Catshark’s eggs hanging from the ramifications of *L*. *glaberrima*. **N.** Specimen of *Scyliorhinus canicula* moving among the coral colonies. **P.** A pair of the crab *Anamathia rissoana* living on a coral colony. **Q.** Entrapped specimen of *Pelagia noctiluca* on a coral colony. Scale Bar: 10 cm.

The bottom of the Carloforte Shoal hosted a plurispecific megabenthic assemblage with almost 1670 specimens of 8 target coral species counted in the examined frames (Fig. 4): the alcyonaceans *Callogorgia verticillata* (Pallas, 1766), *Acanthogorgia hirsuta* Gray, 1857, *Bebryce mollis* Philippi, 1842, *Eunicella cavolinii* (Koch, 1887), *Isidella elongata* (Esper, 1788), the antipatharians *L*. *glaberrima*, *P*. *larix*, and *Antipathes dichotoma* Pallas, 1766.

**Fig 5:**
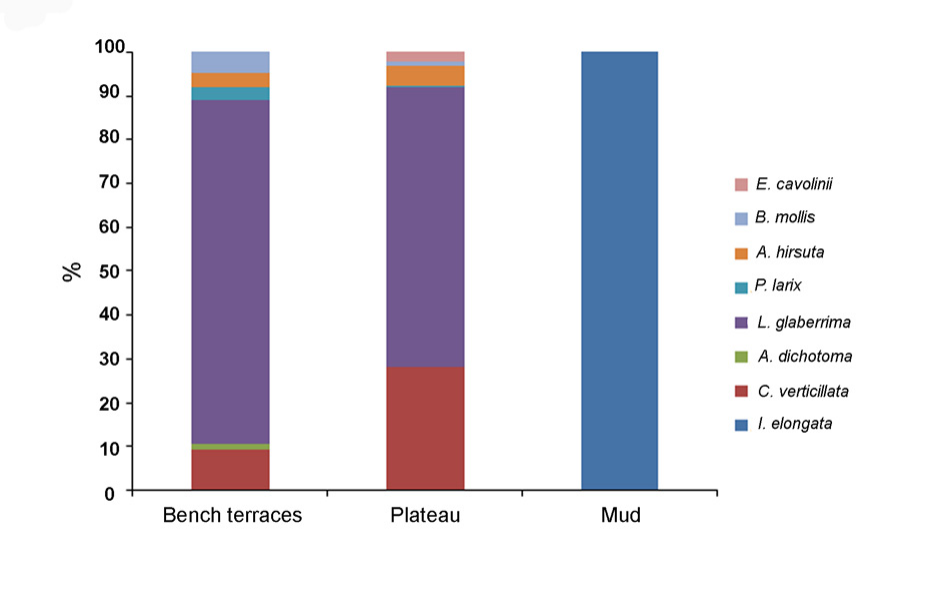
Relative abundance (expressed as %) of the target coral species for the three investigated habitats present on the Carloforte Shoal.

**Fig 6:**
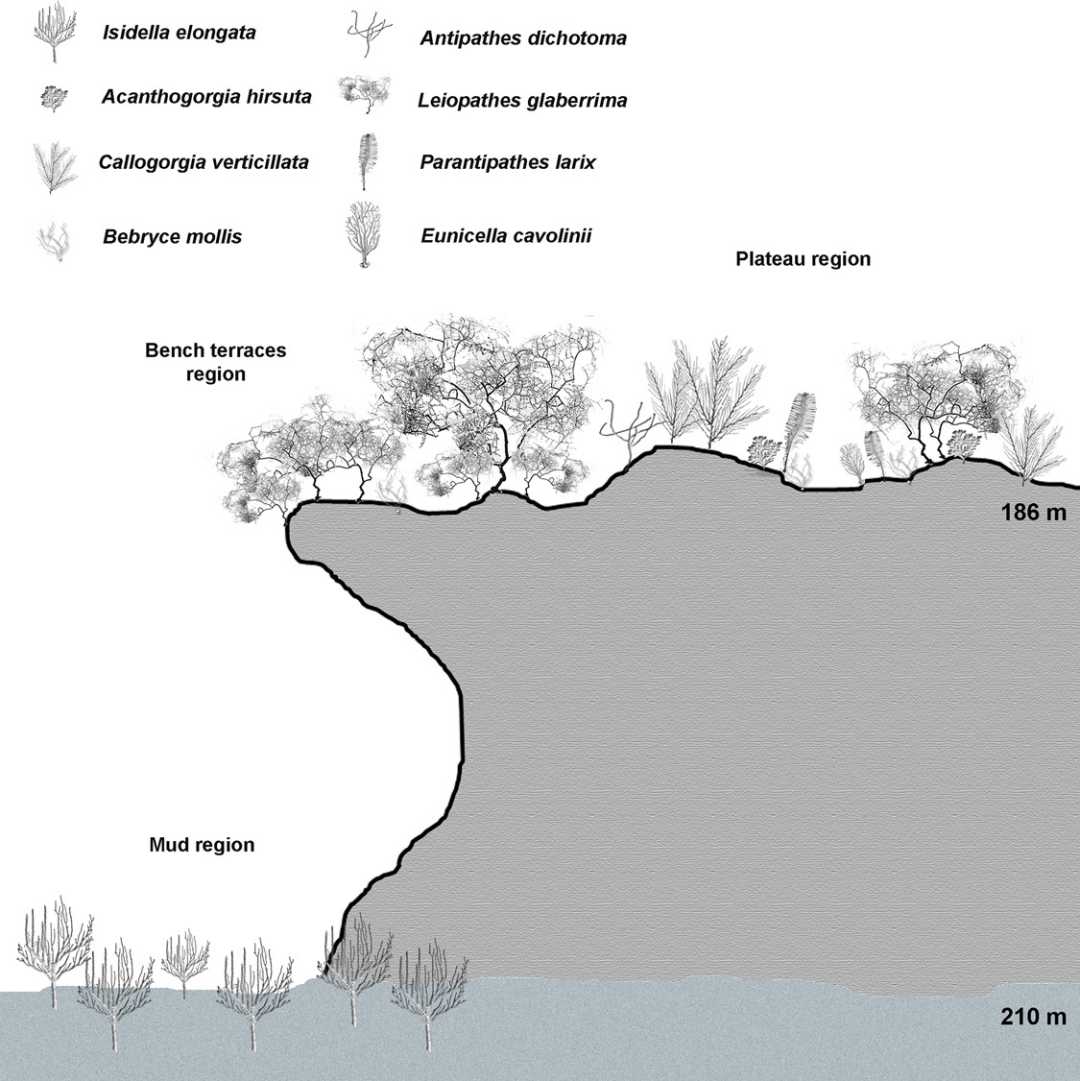
Zonation of the megabenthic assemblages. The Fig. presents the distribution of the most conspicuous and abundant components of the megafauna dwelling between 186–210 m depth on the Carloforte Shoal. Three distinct facies are well represented, one dominated by *Leiopathes glaberrima* forming dense gardens on the Southwestern bench terraces, one dominated by small patches of *L*. *glaberrima* and *Callogorgia verticillata* found on the sparse boulders along the plateau, and one formed by the candelabrum-shaped alcyonacean *Isidella elongata* living on the surrounding muddy floor.

The three considered habitats showed significative differences in terms of species relative abundance (Figs. 5, 6) (ANOSIM, p<0.0001, R 0.26). The bench terraces bordering the Southwestern side of the shoal were dominated by the antipatharian *L*. *glaberrima* (almost 1000 specimens, 79% of the total counted target organisms for this area with an average density of 0.8 ± 0.05 colonies m^-2^) (Figs. 4A-C, 6). In this habitat, the community hosted also *C*. *verticillata* (9%), *B*. *mollis* (5%), *A*. *hirsuta* (3%), *P*. *larix* (3%) and *A*. *dichotoma* (1%) with densities ranging from 0.02 to 0.13 colonies m^-2^. Moreover, the massive sponge *Pachastrella monilifera* Schmidt, 1868 counted here almost 630 specimens for an average density of 0.5 ± 0.04 specimens m^-2^.

In the second habitat, the rocky, silted plateau, the coral community was dominated by a mixed assemblage of *L*. *glaberrima* and *C*. *verticillata* (64% and 28%, respectively). These two species showed significatively lower abundances than on the bench terraces (0.6 ± 0.11 colonies m^-2^ and 0.3 ± 0.07 colonies m^-2^, respectively) (Kruskal-Wallis p<0.0001, H = 112.2 and p<0.001, H = 11.4, respectively) (Figs. 4D-G, 6). The community included also *A*. *hirsuta* (4%), *E*. *cavolinii* (2%) and *B*. *mollis* (1%), with abundances lower than 0.05 colonies m^-2^. Moreover, about 100 specimens of *P*. *monilifera* were recorded, corresponding to 0.35 ± 0.05 colonies m^-2^, significatively lower than on the bench terraces (Kruskal-Wallis, p<0.0001, H = 66.3).

The muddy bottom surrounding the rocky elevations hosted a monospecific population of the candelabrum-shaped alcyonacean *I*. *elongata* (212 colonies, on average 0.5 ± 0.04 colonies m^-2^, with a maximum of 2.7 colonies m^-2^) that has never been reported in the other two habitats (Kruskal-Wallis p<0.0001, H = 133.8) (Figs. 4H-J, 6).

Overall, the total coral abundance is significatively lower in the muddy area (0.5 ± 0.04 colonies m^-2^), than along the plateau and on the bench terraces (1.0 ± 0.12 and 1.1 ± 0.07 colonies m^-2^, respectively) (Kruskal-Wallis p<0.0001, H = 23.25, [M<P = BT]).

In general, relevant traces of fishing impact were not found, with only 2.2% of the total video frames presenting lost gears, exclusively long-lines. All impact traces were observed on the rocky bottoms, especially on the plateau, while no traces were reported on the muddy belt surrounding the base of the explored bench terraces. Only eleven colonies of *L*. *glaberrima* and *C*. *verticillata* were observed evidently entangled in the lines.

### The population of *Leiopathes glaberrima*

**Fig 7:**
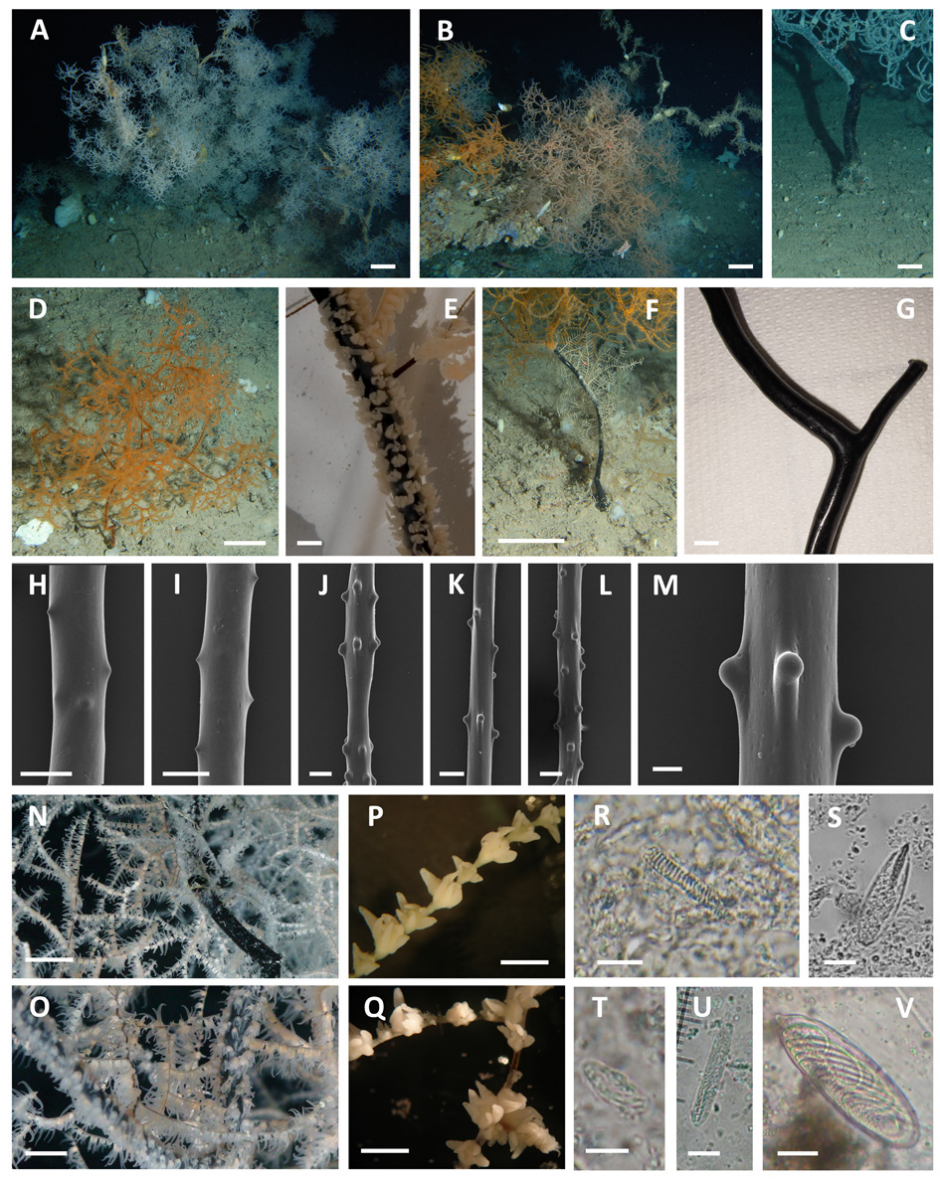
*Leiopathes glaberrima*. **A-B.** Various phenotypes of *L*. *glaberrima* co-occurring in the same population as patches on the rocky outcrops. **C.** Naked basal portion of the stem of the largest colony found in the study area. **D.** Small orange juvenile. **E.** Polyps arrangement along a major branch. **F.** Hydroids colonizing the basal portion of the stem. **G.** Smooth, polished stem (SARD1). **H-M.** Close-up views of the conical spines in branchlets of various diameter (SARD19a). **N-O.** Patches of light orange fertile polyps among white non fertile polyps. **P-Q.** Monoserial arrangement of the polyps along the branchlets. Alternation of adult and juvenile polyps is visible (LEIO). Cnidome (SARD19a, b, c): **R.** Spirocyst. **S.** p-mastigophore. **T.** Small isorhiza. **U.** Basitrich isorhiza. **V.** Large Penicilli E. Scale Bar: A, B, C, D, F: 10 cm; N: 1.5 cm; O: 1 cm; E, G, P, Q: 3 mm; H, I: 0.5 mm; J, K, L: 0.4 mm; M: 0.05 mm; R, S, U: 10 μm; T, V: 5 μm.

The *L*. *glaberrima* specimens of the Carloforte Shoal are characterized by an arborescent corallum sparsely branched, with a relatively long, irregularly arranged terminal branchlets, that are typically 1–5 cm long, with a 0.2–0.5 mm basal diameter (Figs. 4A-D, K; 7A-B) (Table 4).

**Table 4:** Characteristics of ***Leiopathes glaberrima*** from the Carloforte Shoal.

Colony	Height range	Width range	Basal diameter	Branchlets arrangement	Height branchlets spines	Polyps colour	Polyps diameter	Polyps density	Notes
Arborescent	13–210 cm (n = 155)	12–350 cm (n = 155)	0.2–6.8 mm (n = 155)	Irregular	0.04–0.06 mm	White and orange	2–2.4 mm	4–5 polyps cm^-1^	Fertile female polyps light orange

The colonies are bright orange or white (Fig. 7A, D). There is a third phenotype, light orange, whose coloration is likely given by the presence of mature eggs in the polyps (Fig. 7B, N-O). In the investigated population, about half the specimens were white, followed by 30% of light orange colonies and only about 15% of bright orange ones. In large colonies, a complete coenenchymal layer is often lacking on the basal portion of the stem and on the anchorage, which may be covered by other organisms (Fig. 7C-F).

The skeleton of the branchlets presents small, conical spines (0.04–0.06 mm high), organized in 3–4 longitudinal rows, and spaced apart 0.6–1.4 mm (on average 1–2 spines mm^-1^) (Fig. 7H-M). Spines are acute absent on the stem and branches (Fig. 7G). Adult polyps are radial, have a notable size (2–2.4 mm in transverse diameter with sagittal tentacles up to 3.5 mm long when fixed and up to 5.5 mm long alive), and are organized in a single row on the branchlets less than 1.5 mm in diameter (interpolypar distance 0.8–1.2 mm, density of 4–5 polyps cm^-1^) (Fig. 7N-Q). Alternation among adult and juvenile polyps (1–1.2 mm in diameter) is common on these ramifications. Polyps are arranged in two sub-opposite rows on the branchlets 1.5 mm in diameter, and they may aggregate in their intersections (Fig. 7Q). On the contrary, polyps around the stem and major ramifications are irregularly distributed (Fig. 7E). In this latter case, they all have a smaller size (1.6 mm in diameter and may reach densities of 20 polyps cm^-1^). Fertile polyps are distributed in patches along the branchlets: each fertile polyp contains less than 50 gametes present at various stages of maturation with sperm cysts up to 230 μm in diameter, while eggs up to 260 μm in diameter.

Cnidome comprehends spirocysts (abundant in tentacles and mouth) (Fig. 7R), p-mastigophores (few in all regions) (Fig. 7S) and various isorhizae (Type 1: very rare; Type 2: exceptionally abundant in tentacles) (Fig. 7T-U) including a peculiar large one (Type 3: very abundant in tentacles and mesenteries) (Fig. 7V) (Table 5).

**Table 5:** Cnidome of *Leiopathes glaberrima*. Measures of length and width (expressed in μm) are provided for each category.

Cnidome (n = 50)	Spirocysts length	Spirocysts width	p-mastigophores length	p-mastigophores width	Isorhiza T1 length	Isorhiza T1 width	Isorhiza T2 length	Isorhiza T2 width	Isorhiza T3 length	Isorhiza T3 width
Average	24.9	2.9	29.7	8.9	8.8	2.9	28.3	6.0	27.8	10.1
SD	3.09	0.22	0.68	0.21	0.42	0.22	1.74	0.25	1.10	0.10
CV	0.12	0.07	0.02	0.02	0.05	0.08	0.06	0.04	0.04	0.01

SD, standard deviation, CV, coefficient of variation.

In the Carloforte Shoal, *L*. *glaberrima* showed a typical heterogeneous distribution, with colonies aggregated in small patches, particularly along the bench terraces (Figs. 4A-C, 6). The colonies usually displayed a vertical development, whether some were observed also inclined, but never over-hanging. Twenty-one distinct patches were counted at a short distance from each other’s along both the terraces and the plateau, comprising 7 to 186 colonies, distributed on a surface ranging from 5 to 83 m^2^. In the patches, mainly corresponding each one to a distinct rocky terrace or rocky emergence, *L*. *glaberrima* showed an average abundance of 1.4 ± 0.8 colonies m^-2^ (ranging from 0.3 to 2.5 colonies m^-2^). Peaks of about 8 colonies m^-2^ were observed in some frames. Considering the total number of counted colonies in the frames, it is possible to hypothesize the occurrence of over 2600 black coral colonies along the entire recorded video transects.

The arborescent skeleton of *L*. *glaberrima* may represent a substrate for the development of numerous encrusting organisms, in particular hydroids (Sertulariidae), sponges, bryozoans, zoanthids, and polychaetes living on the dead portions of the colonies (Fig. 7F). Occasionally it is possible to observe, on the naked branchlets, ophiuroids and crinoids, gastropods, Anomiidae bivalves, and, more rarely, small anthozoans such as *A*. *hirsuta* and *B*. *mollis*. No close symbiotic associations have been recorded with the living portions of the corals (which may produce a great amount of mucous when disturbed) with the exception of the crab *Anamathia rissoana* (Roux, 1828) (35 specimens counted), often observed in a pairs on the colonies (Fig. 4P). Notable is a 2 m tall specimen (Fig. 4K) hosting at least 8 crabs within its ramifications. Various fish species find a temporary refuge in the coral, such as *Anthias anthias* (Linnaeus, 1758), *Macroramphosus scolopax* (Linnaeus, 1758), *Zeus faber* Linnaeus, 1758, *Lappanella fasciata* (Cocco, 1833), *Trachurus* sp., and *Benthocometes robustus* (Goode & Bean, 1886), the latter being one of the most characteristic of this coral species. A deep-sea school of the jellyfish *Pelagia noctiluca* (Forskål, 1775), with 51 specimens counted entrapped in the ramifications of the corals (Fig. 4Q), was reported in the sampling of 2013.

Eight specimens of the catshark *Scyliorhinus canicula* (Linnaeus, 1758) were observed in the area, moving on the muddy bottom near the rocky elevations and within the coral branches (Fig. 4O). This species uses the ramifications of the black coral colonies to lay the eggs. A total of 743 capsules, at different stages of maturation, were observed on the colonies (Fig. 4L-N), with some corals lacking eggs and one, 2 m tall, hosting 44 capsules. One skate’s egg was also observed hanging on the same coral.

### Population structure and age of the black coral colonies

**Fig 8:**
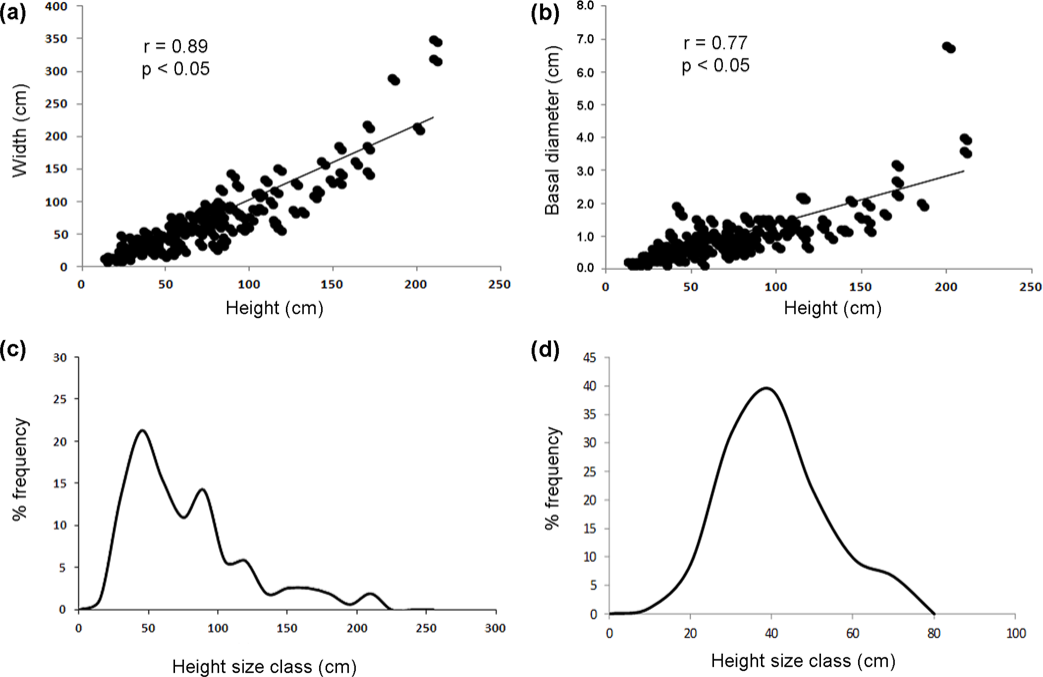
Linear regression of sizes (a) and basal diameter (b) in *Leiopathes glaberrima*. Size (height)-frequency distribution in *L*. *glaberrima* (**c**) and *Isidella elongata* (**d**).

A total of 155 colonies of *L*. *glaberrima* were present in the video frames in complete frontal view and therefore were considered suitable for a morphometric study. The analyzed colonies had an average height of 70 ± 3.4 cm and an average width of 69 ± 4.4 cm with a regular isometric growth (Fig. 8a) (Pearson’s r = 0.89, p<0.05). Three very large specimens (with heights and widths exceeding 200 cm) were recorded, characterized by a thin, fan-like profile. The basal diameter of the stem, on average 1.0 ± 0.1 cm, is linearly correlated to the height of the colony (Pearson’s r = 0.77, p<0.05), even if there are few large specimens with exceptionally large diameter (maximum of 6.8 cm in a colony 200 cm high and with a 4 m^2^ of surface) (Figs. 4K; 7C; 8b).

The size frequency distribution of the heights is a curve with a maximum peak in the class of 45–50 cm. The population, however, shows also other three minor peaks, corresponding to the size classes 90–95, 120–125 and 160–165 cm and a long tail of colonies over 200 cm of height (Fig. 8c).

Data for the conventional radiocarbon dating carried out on a sample of *L*. *glaberrima* are listed in Table 3. Calibrated ages are also reported with a quoted uncertainty corresponding to 68.2% of confidence level. The analysis of the inner part of the base of *L*. *glaberrima* (diameter of 4 cm) from the study area was thus dated to 40 ± 55 AD (Anno Domini), which corresponds to an age of 1973 years before the date of the test (2013).

### Sandy bottoms: population structure of *Isidella elongata*

The muddy, flat bottom surrounding the rocky terraces of the Carloforte Shoal at about 210 m depth, hosted a rich population of *I*. *elongata* (Figs. 4H-J, 6). Colonies, displaying the typical candelabrum-shaped morphology with a clear alternation of white carbonatic internodes and brown organic nodes along the ramifications [53], hosted no associated megafauna.

The population structure of *I*. *elongata*, based on 109 analyzed specimens, reveals a distinct peak for the size class 40 cm high with a good representation of the juvenile size classes (Fig. 8d). On average the colonies are 36 ± 1.2 cm tall (maximum 66 cm) and are 22 ± 1.0 cm large (maximum 55 cm) with a regular isometric growth (Spearman’s rho = 0.65, p<0.001).

## Discussion

Few studies have been conducted in the Mediterranean Sea to assess the impact of fishing activities on the benthic communities including long-lived marine species and they were mainly focused on the soft bottoms assemblages affected by trawlers [54, 55]. Recent ROV data suggest that also the coral communities living on rocky bottoms and acting as true oases of biodiversity [8], are directly and indirectly impacted by fishing activities [11, 15]. A recent study found that during experimental long-line fishing activities carried out between 500–600 m depth in the eastern Mediterranean Sea using hooks targeting hake and blackspot seabream, a major coral bycatch occurred, with Antipatharia being the most frequently caught coral group [16]. Living cold water corals occurred in 72% of the long lines with an annual bycatch estimated to be of approximately 30 colonies of *L*. *glaberrima* per fisherman. High long lines bycatch values for *Leiopathes* species have been reported also in the Azores [56]. Differences are probably related to the abundance of the coral species in the area, to the catchability of the gear, to the morphological structure of the coral [16] and to its mechanical resistance [11]. In consideration of these data, therefore, so far, pristine black coral ecosystems can be considered rare in the Mediterranean basin as already stated for precious red coral populations [18].

This work focused on a rich deep-sea coral community of Sardinia, an area well known for its rich red coral banks [57, 58], but yet poorly explored for what concerns the other components of the deep-sea assemblages. The population of the Carloforte Shoal is the largest ever described so far for *L*. *glaberrima* with an estimation of over 2600 colonies in the sole explored area. This species shows a clear habitat preference, along the rocky bench terraces, which is in agreement with previous observations made along the Sicilian margin where the colonies seemed to favor elevated positions probably in accordance with local current regimes [15]. The colonies show a patchy distribution typical of all arborescent anthozoans characterized by a limited larval dispersion [12, 59, 60]. Patches typically show a high density of colonies such that the occurrence of few sparse colonies with traces of damage has been considered as a bioindicator of impacted site [11].

This species, due to its arborescent corallum and its tendency of forming dense aggregations, fits in the definition of habitat-forming species, an organism able to three-dimensionally structure the environment and enhance its ecological functionality [3]. The black coral population of the Carloforte Shoal is inhabited by a huge variety of sessile and vagile organisms, searching for a refuge or a source of food, supporting the hypothesis that the existence of the coral canopy is able to enhance biodiversity at every level [4, 61]. The long list of sessile organisms observed here associated to *L*. *glaberrima* misses only epibiontic colonies of the scleractinians *Madrepora oculata* Linnaeus, 1758, *Lophelia pertusa* (Linnaeus, 1758) and *Desmophyllum dianthus* (Esper, 1794) occasionally reported on dead branches of deeper colonies [22]. Moreover, similarly to other biological structures made of large anthozoans [62], the habitat formed by *L*. *glaberrima* hosts a nursery area [63]. Despite *S*. *canicula* may locally represent a common component of fishing stocks in the Mediterranean Sea [64], it is not considered a threatened species [54]. However, it is evidently crucial the close dependency among the survivorship of local populations of catsharks with the existence of the coral colonies supporting their eggs. Finally, the record of numerous jellyfish *P*. *noctiluca* within the coral ramifications is in agreement with the supposed late summer-autumn vertical migration of this species recently hypothesized for nutrients-rich areas, such as canyons [65]. The jellyfish in the Carloforte Shoal were recorded entrapped within the coral ramifications with tentacles clearly entangled by the polyps, suggesting that gelatinous zooplankton may occasionally be a food source for deep-sea coral species.

This work describes for the first time the cnidome of *L*. *glaberrima*, characterized by the presence of an un-usually large isorhiza. This nematocyst closely resembles in shape the large penicilli E (16–26 x 9–10 μm) identified for the zoanthid *Savalia savaglia* (Bertoloni, 1819) by [66], whether no data on the ultrastructural ornamentation of the filament is available for *L*. *glaberrima*. This isorhiza is not the largest known for black corals: *Pseudocirrhipathes mapia* Bo & Bavestrello, 2009 and the related species *Allopathes desbonni* (Duchassaing & Michelotti, 1864) and *Aphanipathes verticillata* Brook, 1889 are known to possess a large basitrich isorhiza (35 x 5 μm) possessing a strong taxonomic value [67].

This work describes for the first time also the structure of a population of *L*. *glaberrima*. In the Mediterranean Sea, this datum was mainly reported for shallow waters alcyonaceans [6, 68], and, among black coral species, it was elucidated only for *A*. *subpinnata* and *P*. *larix* [2, 12]. In the first case, the demography of the Favazzina black coral population showed a peak in the smaller size classes, interpreted as an indicator of a young population supported by a high settling rate [2]. A lack of *L*. *glaberrima* juvenile colonies on the Carloforte Shoal can partially be ascribed to the difficulty in identifying small colonies among arborescent adults in the video frames. However, in consideration of the high number of observed colonies, it is plausible that the population is well represented by at least four distinct cohorts of colonies attributable to a discontinuous recruitment and that the low abundance of juveniles may reflect an overall stable population of a slow growing organism.

For the first time the presence of millennial organisms was described in the Mediterranean Sea. Comparable ages were already reported for *Leiopathes* species from deeper oceanic regions [27, 35, 26, 28], but the Sardinian record additionally suggests a millennial stability of a deep-sea biocoenosis in a semi-enclosed, heavily exploited basin. Considering results of radiocarbon dating and the population size structure, it is probable that in the Carloforte Shoal’s garden a major proportion of the colonies is hundreds years old with few millennial specimens. The stability of the environment and its biocoenoses is supported also by the occurrence of a large meadow of *I*. *elongata*, another deep-sea anthozoan showing slow growth rates [69] and a long life span (400 years) [70].

It seems highly probable that the persistence of the Carloforte Shoal biocoenosis is due to the rough topography of the region characterized by numerous rocky elevations sufficiently high to avoid trawling and limiting long line fishing (Fig. 1b). This situation preserves both the rocky communities and the population of *I*. *elongata* surviving in the small enclaves of soft bottom nearby the rocks. Due to its occurrence on incoherent grounds populated by commercial shrimps, this alcyonacean was, among the deep-sea species, one of the most commonly reported in the past as part of the coral bycatch [71]. Known to form characteristic facies in the bathyal muds of some areas of the western Mediterranean basin, *I*. *elongata* has been reported also along the western Sardinian platform slope [53]. However, at present, there are numerous indications of a substantial constriction of the areal of this species in various Mediterranean highly trawled fishing grounds [71, 72, 55, 73] as well as in artisanal fishing areas [16] and, today, it is considered rare. Small enclaves of *I*. *elongata* may therefore survive in the shallowest or deepest ranges of the species distribution, for example in shallow water refuges of muddy bottoms protected by trawling activities (as in the Sardinian case) or in grounds where trawling is forbade, for example on slope grounds or below 1000 m depth [73].

In the last decades, the Mediterranean shallow waters have experienced deep changes generally attributed to anthropogenic pollution and global warming [74]. These changes resulted in impressive mass mortality events and quali-quantitative modifications in the composition of benthic communities [75]. On the other hand, past investigations have always suggested a high stability of the Mediterranean bathyal ecosystems [76], even if more recent studies suggested that some modifications, due to climate changes, are influencing the quantity and quality of food reaching deep-sea ecosystems [77]. The persistence, on a millennial span of time, of the Carloforte Shoal coral population in a supposedly pristine state suggests that these variations, along the deep continental platform, may have only a limited effect on these off-shore deep-sea coral communities that, on the contrary, are very sensitive to fishing impact. Effects of fishing activities on the seabed and benthic communities have been indeed compared to those of forest clear-cutting, with an immediate reduction of the structural diversity and following alteration of biogeochemical cycles, species recovery and settling rates [78]. Effects are more severe when fishing frequency is high, especially in deep-sea ecosystems, where natural disturbance is limited and biological processes are slow [78].

Black coral gardens represent long-term biological archives of inestimable value [29, 27, 28] and, despite the fact that new assemblages are found more frequently than what expected in the past, the consequences of their destruction is amplified by their low recovery ability due to natural fragmentation of the populations [12], slow growth rates [26], limited larval dispersion and population connectivity [59], late maturity age [79], susceptibility of the colonies to impacts and habitat destruction [11].

## Conclusions

Coral aggregations have now been internationally identified as special ecological features that require protection under the Convention of Biological Diversity [80]. More specifically, “Coral Garden” habitats, such as those formed by black corals on rocky shoals and by *I*. *elongata* on compact muds, were added to the OSPAR (Oslo and Paris Conventions for the Protection of the Marine Environment of the North-East Atlantic) “List of threatened and/or declining species and habitats” in 2007 [80]. Due to the fact that they are considered sensitive habitats, potentially impacted by deep-sea fisheries, they may be identified as Vulnerable Marine Ecosystems (VMEs). Recently, the Food and Agriculture Organization (FAO) [81] recommended the establishment of protected areas where such VMEs are known to be or likely to occur in order to put into action an ecosystem-based fishery management of deep-sea ecosystems, as recently carried out in the Gulf of Lion [42]). Despite the European Commission has now proposed regulations against the use of trawling nets on important ecosystems as coralligenous, seamounts and white coral mounds [82], in the Mediterranean waters, the interdiction of these deep-sea coral sanctuaries located on trawling routes or within traditional artisanal fishing grounds, would raise numerous socio-economic problems.

## Supporting Information

S1 Dataset Matrix of raw data and results of statistical analyses. (XLS)

## References

[1] Food and Agriculture Organization (FAO). Global forest resources assessment 2005. Progress towards sustainable forest management. FAO Forestry Paper, Rome. 2006; 147: 350 pp.

[2] Bo M, Bavestrello G, Canese S, Giusti M, Salvati E, Angiolillo A, et al Characteristics of a black coral meadow in the twilight zone of the central Mediterranean Sea. Mar Ecol Prog Ser. 2009; 397: 53–61.

[3] Buhl-Mortensen L, Vanreusel A, Gooday AJ, Levin LA, Priede IG, Buhl‐Mortensen P, et al Biological structures as a source of habitat heterogeneity and biodiversity on the deep ocean margins. Mar Ecol. 2010; 31: 21–50.

[4] Cerrano C, Danovaro R, Gambi C, Pusceddu A, Riva A, Schiaparelli S. Gold coral (*Savalia savaglia*) and gorgonian forests enhance benthic biodiversity and ecosystem functioning in the mesophotic zone. Biodiv Conserv. 2010; 19: 153–167.

[5] Bo M, Tazioli S, Spanò N, Bavestrello G. *Antipathella subpinnata* (Antipatharia, Myriopathidae) in Italian seas. It J Zool. 2008, 75: 185–195.

[6] Gori A, Rossi S, Berganzo E, Pretus JL, Dale MRT, Gili JM. Spatial distribution patterns of the gorgonians *Eunicella singularis*, *Paramuricea clavata*, and *Leptogorgia sarmentosa* (Cape of Creus, Northwestern Mediterranean Sea). Mar Biol. 2011, 158: 143–158.

[7] Gori A, Rossi S, Linares C, Berganzo E, Covadonga O, Dale MR, et al Size and spatial structure in deep versus shallow populations of the Mediterranean gorgonian *Eunicella singularis* (Cap de Creus, northwestern Mediterranean Sea). Mar Biol. 2011, 158: 1721–1732.

[8] Bo M, Canese S, Spaggiari C, Pusceddu A, Bertolino M, Angiolillo M, et al Deep Coral Oases in the South Tyrrhenian Sea. PLoS-One. 2012, 7: e49870 10.1371/journal.pone.0049870 23185468 PMC3503811

[9] Weinberg S. Mediterranean octocoral communities and the abiotic environment. Mar Biol. 1978, 49: 41–57.

[10] Bo M, Bertolino M, Borghini M, Castellano M, Covazzi Harriague A, Di Camillo CG, et al Characteristics of the mesophotic megabenthic assemblage of the Vercelli Seamount (North Tyrrhenian Sea). PLoS-One. 2011, 6: e16357 10.1371/journal.pone.0016357 21304906 PMC3033400

[11] Bo M, Bava S, Canese S, Angiolillo M, Cattaneo-Vietti R, Bavestrello G. Fishing impact on deep Mediterranean rocky habitats as revealed by ROV investigation. Biol Conserv. 2014, 171: 167–176.

[12] Bo M, Canese S, Bavestrello G. Discovering Mediterranean black coral forests: *Parantipathes larix* (Anthozoa: Hexacorallia) in the Tuscan Archipelago. Italy It J Zool. 2014, 81: 112–125.

[13] Vogel S. Life in Moving Fluids: the physical biology of flow. Princeton University Press Princeton, New Jersey, USA 1994: 467 pp.

[14] Ponti M, Perlini RA, Ventra V, Grech D, Abbiati M, Cerrano C. Ecological shifts in Mediterranean coralligenous assemblages related to gorgonian forest loss. PLoS-One. 2014, 9: e102782 10.1371/journal.pone.0102782 25054286 PMC4108394

[15] Bo M, Cerrano C, Canese S, Salvati E, Angiolillo M, Santangelo G, et al The deep coral assemblages of an off-shore deep Mediterranean rocky bank (NW Sicily, Italy). Mar Ecol. 2014, 35: 332–342. 10.1097/MNM.0000000000000058 24463547

[16] Mytilineou C, Smith CJ, Anastasopoulou A, Papadopoulou KN, Christidis G, Bekas P, et al New cold-water coral occurrences in the Eastern Ionian Sea: results from experimental long line fishing. Deep Sea Res II. 2014, 99: 146–157.

[17] Deidun A, Tsounis G, Balzan F, Micallef A. Records of black coral (Antipatharia) and red coral (*Corallium rubrum*) fishing activities in the Maltese Islands. Mar Biodiv Rec. 2010, 3: e90.

[18] Tsounis G, Rossi S, Grigg R, Santangelo G, Bramanti L, Gili JM. The exploitation and conservation of precious corals Ocean. Mar Biol: Ann Rev. 2010, 48: 161–212.

[19] Opresko DM, Försterra G. Orden Antipatharia (corales negros o espinosos) In Hofrichter R ed. El Mar Mediterraneo: Fauna, Flora, Ecologia, Vol 2 Omega, Barcelona, 2004, 506–509.

[20] Bo M, Bavestrello G, Canese S, Giusti M, Angiolillo M, Cerrano C, et al Coral assemblages off the Calabrian Coast (South Italy) with new observations on living colonies of *Antipathes dichotoma* . It J Zool. 2010, 78: 231–242.

[21] D’Onghia G, Maiorano P, Sion L, Giove A, Capezzuto F, Carlucci R, et al Effects of deep-water coral banks on the abundance and size structure of the megafauna in the Mediterranean Sea. Deep Sea Res II. 2010, 57: 397–411.

[22] Angeletti L, Taviani M, Canese S, Foglini F, Mastrototaro F, Argnani A, et al New deep-water cnidarian sites in the southern Adriatic Sea. Medit Mar Sci. 2014, 15: 263–273.

[23] Fabri MC, Pedel L, Freiwald A, Madurell T. Habitats particuliers des étages bathyal et abyssal. SRM MO Initial Assessment for the Water Marine Framework Strategy. 2012. Available from https://wwwifremerfr/sextant_doc/dcsmm/documents/Evaluation_initiale/caracteristiques_etat_ecologique/MED/EE_110621_Habitats-particuliers-bathyalV3_MO_2012.

[24] Druffel ER, Griffin S, Witter A, Nelson E, Southon J, Kashgarian M, et al *Gerardia*: bristlecone pine of the deep-sea? Geo Cosmo Acta. 1995, 59: 5031–5036.

[25] Roark E, Guilderson TP, Dunbar RB, Ingram B. Radiocarbon-based ages and growth rates of Hawaiian deep-sea corals. Mar Ecol Prog Ser. 2006, 327: 1–14.

[26] Prouty NG, Roark EB, Buster NA, Ross SW. Growth rate and age distribution of deep-sea black corals in the Gulf of Mexico. Mar Ecol Prog Ser. 2011, 423: 101–115.

[27] Roark EB, Guilderson TP, Dunbar RB, Fallon SJ, Mucciarone DA. Extreme longevity in proteinaceous deep-sea corals. Proc Nat Acad Sci USA. 2009, 106: 5204–5208. 10.1073/pnas.0810875106 19307564 PMC2663997

[28] Carreiro-Silva M, Andrews AH, Braga-Henriques A, de Matos V, Porteiro FM, Santos RS. Variability in growth rates of long-lived black coral *Leiopathes* sp. from the Azores. Mar Ecol Prog Ser. 2013, 473: 189–199.

[29] Williams B, Risk MJ, Ross SW, Sulak KJ. Deep-water antipatharians: proxies of environmental change. Geol. 2006, 34: 773–776.

[30] Williams B, Risk MJ, Ross SW, Sulak KJ. Stable isotope data from deep-water antipatharians: 400-year records from the southeastern coast of the United States of America. Bull Mar Sci. 2007, 81: 437–447.

[31] Risk MJ, Sherwood OA, Nairn R, Gibbons C. Tracking the record of sewage discharge off Jeddah, Saudi Arabia, since 1950, using stable isotope records from antipatharians. Mar Ecol Prog Ser. 2009, 397: 219–226.

[32] Raimundo J, Vale C, Caetano M, Anes B, Carreiro-Silva M, Martins I, et al Element concentrations in cold-water gorgonians and black coral from Azores region. Deep Sea Res II. 2013, 98: 129–136.

[33] Opresko DM. Three new species of *Leiopathes* (Cnidaria: Anthozoa: Antipatharia) from Southern Australia. Rec S Aust Mus. 1998, 31: 99–111.

[34] Molodtsova TN. A new species of *Leiopathes* (Anthozoa: Antipatharia) from the Great Meteor seamount (North Atlantic). Zootaxa. 2011, 3138: 52–64.

[35] Opresko DM, Baron-Szabo RC. Re-descriptions of the antipatharians corals described by EJC Esper with selected English translations of the original German text (Cnidaria, Anthozoa, Antipatharia). Senckenberg Biol. 2001, 81: 1–21.

[36] Vafidis D, Mytilineou C, Mastrototaro F, D'Onghia G. First records of *Leiopathes glaberrima* (Esper, 1792) and *Isidella elongata* (Esper, 1788) (Cnidaria: Anthozoa) in the Ionian Sea. Proceedings of the 10th ICZEGAR, Patra, Greece. 2006, 220 p.

[37] Sinniger F, Pawlowski J. The partial mitochondrial genome of *Leiopathes glaberrima* (Hexacorallia: Antipatharia) and the first report of the presence of an intron in COI in black corals Galaxea. J Cor Reef Stu. 2009, 11: 21–26.

[38] Smith C, Sakellariou D, McCoy F, Wachsmann S. Deep coral environments south of Crete 9ο Πανελλήνιο Συμπόσιο Ωκεανογραφίας & Αλιείας 2009—Πρακτικά, Τόμος. 2009, 1: 665–668.

[39] Mastrototaro F, D’Onghia G, Corriero G, Matarrese A, Maiorano P, Panetta P, et al Biodiversity of the white coral and sponge community off Cape Santa Maria di Leuca (Mediterranean Sea): un update. Deep Sea Res II. 2010, 57: 412–430.

[40] Vertino A, Savini A, Rosso A, Di Geronimo A, Mastrototaro F, Sanfilippo R, et al Benthic habitat characterization and distribution from two representative sites of the deep-water SML Coral Province (Mediterranean). Deep Sea Res II. 2010, 57: 380–396.

[41] Pardo E, Aguilar R, García S, de la Torriente A, Ubero J. Documentación de arrecifes de corales de agua fría en el Mediterráneo occidental (Mar de Alborán). Chro. Nat. 2011, 1: 20–34.

[42] Fabri MC, Pedel L, Beuck L, Galgani F, Hebbeln D, Freiwald A. Megafauna of vulnerable marine ecosystems in French Mediterranean submarine canyons: Spatial distribution and anthropogenic impacts. Deep Sea Res II. 2014, 104: 184–207.

[43] Olita A, Ribotti A, Fazioli L, Perilli A, Sorgente R. Surface circulation and upwelling in the Sardinia Sea: A numerical study. Cont Shelf Res. 2013, 71: 95–108.

[44] Hammer Ø, Harper DAT, Ryan PD. PAST: paleontological statistics software package for education and data analysis. Pal Electro. 2001, 4: 1–9.

[45] Calcagnile L, Quarta G, D'Elia M, Rizzo A, Gottdang A, Klein M, et al A new accelerator mass spectrometry facility in Lecce, Italy. Nucl Instr Met Phy Res Sec B: Beam Inter Mat Atoms. 2004, 223: 16–20.

[46] Longin R. New method of collagen extraction for radiocarbon dating. Nature. 1971, 230: 241–242. 4926713 10.1038/230241a0

[47] Gianfrate G, D’Elia M, Quarta G, Giotta L, Valli L, Calcagnile L. Qualitative application based on IR spectroscopy for bone sample quality control in radiocarbon dating. Nuc Instr Met Phy Res Sec B: Beam Inter Mat Atoms. 2007, 259: 316–319.

[48] D'Elia M, Calcagnile L, Quarta G, Sanapo C, Laudisa M, Toma U, et al Sample preparation and blank values at the AMS radiocarbon facility of the University of Lecce. Nuc Instr Met Phy Res Sec B: Beam Inter Mat Atoms. 2004, 223: 278–283.

[49] Stuiver M, Polach HA. Discussion: reporting of 14C data. Radiocarbon. 1977, 19: 355–363.

[50] Reimer PJ, Baillie MGL, Bard E, Bayliss A, Beck JW, Blackwell PG, et al IntCal09 and Marine09 radiocarbon age calibration curves, 0–50,000 years cal BP. Radiocarbon. 2009, 51, 1111–1150.

[51] Bronk Ramsey C. Development of the radiocarbon calibration program. Radiocarbon. 2001, 43: 355–263.

[52] Reimer PJ, McCormac FG. Marine radiocarbon reservoir corrections for the Mediterranean and Aegean sea. Radiocarbon. 2002, 44: 159.

[53] Carpine C, Grasshoff M. Les gorgonaires de la Méditerranée. Bull Inst Océan Monaco. 1973, 71: 1–140.

[54] Maynou F, Sbrana M, Sartor P, Maravelias C, Kavadas S, Damalas D, et al Estimating Trends of Population Decline in Long-Lived Marine Species in the Mediterranean Sea Based on Fishers’ Perceptions. PLOSone. 2011, 6: e21818 10.1371/journal.pone.0021818 21818268 PMC3139578

[55] Maynou F, Cartes JE. Effects of trawling on fish and invertebrates from deep-sea coral facies of *Isidella elongata* in the western Mediterranean. J Mar Biol Ass UK. 2012, 92: 1501–1507.

[56] Sampaio I, Braga-Henriques A, Pham C, Ocaña O, de Matos V, Morato T, et al Cold-water corals landed by bottom longline fishery in the Azores. J Mar Biol Ass UK. 2012, 92: 1547–1555.

[57] Cannas R, Caocci F, Follesa MC, Pedoni C, Pendugiu AA, Pesci P, et al The red coral resource in Sardinian seas: a multidisciplinary survey on *Corallium rubrum* populations. St Tr Sci Nat. 2011, 89: 9–18.

[58] Follesa MC, Cannas R, Cau Al, Pedoni C, Pesci P, Cau A. Deep-water red coral from the island of Sardinia (north-western Mediterranean): a local example of sustainable management Mar Freshw Res. 2013, 64: 706–715.

[59] Miller KJ. Short-distance dispersal of black coral larvae: inference from spatial analysis of colony genotypes. Mar Ecol Prog Ser. 1998, 163: 225–233.

[60] Costantini F, Fauvelot C, Abbiati M. Fine-scale genetic structuring in *Corallium rubrum* (L): evidences of inbreeding and limited effective larval dispersal. Mar Ecol Prog Ser. 2007, 340: 109–119.

[61] Bongiorni L, Mea M, Gambi C, Pusceddu A, Taviani M, Danovaro R. Deep-water corals promote higher diversity in deep-sea meiofaunal assemblages along continental margins. Biol Conserv. 2010, 143: 1687–1700.

[62] Etnoyer P, Warrenchuk J. A catshark nursery in a deep gorgonian field in the Mississippi Canyon, Gulf of Mexico. Bull Mar Sci. 2007, 81: 553–559.

[63] Cau A, Follesa MC, Bo M, Canese S, Bellodi A, Cannas R, et al. *Leiopathes glaberrima* forest from South West Sardinia: a thousand years old nursery area for the small spotted catshark *Scyliorinus canicula*. Rapport Commission International Mer Mediterranee, Marseilles. 2014, p 40.

[64] Damalas D, Vassilopoulou V. Chondrichthyan by-catch and discards in the demersal trawl fishery of the central Aegean Sea (Eastern Mediterranean). Fish Res. 2011, 108: 142–152.

[65] Canepa A, Fuentes V, Sabatés A, Piraino S, Boero F, Gili JM. *Pelagia noctiluca* in the Mediterranean Sea. In Pitt KA, Lucas CH (Eds), Jellyfish Blooms, Springer Netherlands 2014,pp 237–266.

[66] Ocaña O, Brito A. A review of Gerardiidae (Anthozoa: Zoantharia) from the Macaronesian islands and the Mediterranean Sea with the description of a new species. Rev Acad Can Cien. 2004, 15: 159–189.

[67] Bo M, Barucca M, Biscotti MA, Canapa A, Lapian HFN, Olmo E, et al Description of *Pseudocirrhipathes* (Cnidaria: Anthozoa: Hexacorallia: Antipathidae), a new genus of whip black corals from the Indo‐Pacific. It J Zool. 2009, 76: 392–402.

[68] Linares C, Coma R, Garrabou J, Dìaz D, Zabala M. Size distribution, density and disturbance in two Mediterranean gorgonians: *Paramuricea clavata* and *Eunicella singularis* . J Appl Ecol. 2008, 45: 688–699.

[69] Andrews AH, Stone RP, Lundstrom CC, DeVogelaere AP. Growth rate and age determination of bamboo corals from the northeastern Pacific Ocean using refined 210Pb dating. Mar Ecol Prog Ser. 2009, 397: 173–185.

[70] Sherwood OA, Thresher RE, Fallon SJ, Davies DM, Trull TW. Multi-century time-series of 15N and 14C in bamboo corals from deep Tasmanian seamounts: evidence for stable oceanographic conditions. Mar Ecol Prog Ser. 2009, 397: 209–218.

[71] Relini G, Peirano A, Tunesi L. Osservazioni sulle comunità dei fondi strascicatoli del Mar Ligure Centro-Orientale. Boll Mus Ist Biol Univ Genova. 1986, 52: 139–16.

[72] D’Onghia G, Mastrototaro F, Matarrese A, Politou C, Mytilineou C. Biodiversity of the upper slope demersal community in the eastern Mediterranean: preliminary comparison between two areas with and without trawl fishing. J North Atl Fish Sci. 2003, 31: 263.

[73] Cartes JE, LoIacono C, Mamouridis V, López-Pérez C, Rodríguez P. Geomorphological, trophic and human influences on the bamboo coral *Isidella elongata* assemblages in the deep Mediterranean: To what extent does *Isidella* form habitat for fish and invertebrates? Deep Sea Res I. 2013, 76: 52–65.

[74] Bianchi CN, Morri C. Marine biodiversity of the Mediterranean Sea: situation, problems and prospects for future research. Mar Poll Bull. 2000, 40: 367–376.

[75] Cerrano C, Bavestrello G, Bianchi CN, Cattaneo‐Vietti R, Bava S, Morganti C, et al A catastrophic mass‐mortality episode of gorgonians and other organisms in the Ligurian Sea (North‐western Mediterranean), summer 1999. Ecol Lett. 2000, 3: 284–293.

[76] Grassle JF. Slow recolonisation of deep-sea sediment. Lett Nat. 1977, 265: 618–619.

[77] Danovaro R, Dell'Anno A, Fabiano M, Pusceddu A, Tselepides A. Deep-sea ecosystem response to climate changes: the eastern Mediterranean case study. Trends Ecol Evol. 2001, 16: 505–510.

[78] Watling L, Norse EA. Disturbance of the seabed by mobile fishing gear: a comparison to forest clearcutting. Conserv Biol. 1998, 12: 1180–1197.

[79] Parker NR, Mladenov PV, Grange KR. Reproductive biology of the antipatharian black coral *Antipathes fiordensis* in Doubtful Sound, Fiordland, New Zealand. Mar Biol. 1997, 130: 11–22.

[80] Aguiliar R, Marín P. Mediterranean deep-sea corals: reasons for protection under the Barcelona Convention. 2013. Oceana Available from http://oceanaorg/sites/default/files/euo/OCEANA_Brief_Deep-sea_Coralspdf.

[81] Food and Agriculture Organization (FAO). International Guidelines for the Management of Deep-sea Fisheries in the High Seas. FAO, Rome, Italy 2009, p 73.

[82] European Commission. Proposal for a Council Regulation concerning management measures for the sustainable exploitation of fishery resources in the Mediterranean Sea and amending Regulations (EC) No. 2847/93 and (EC) No.973/2001 (COM(2003) 589 final-2003/0229 (CNS)). 2003.

